# HIV Coinfection Provides Insights for the Design of Vaccine Cocktails to Elicit Broadly Neutralizing Antibodies

**DOI:** 10.1128/jvi.00324-22

**Published:** 2022-06-27

**Authors:** Daniel J. Sheward, Tandile Hermanus, Ben Murrell, Nigel Garrett, Salim S. Abdool Karim, Lynn Morris, Penny L. Moore, Carolyn Williamson

**Affiliations:** a Institute of Infectious Diseases and Molecular Medicine, Division of Medical Virology, Department of Pathology, University of Cape Town, Cape Town, South Africa; b Department of Microbiology, Tumor and Cell Biology, Karolinska Institutet, Stockholm, Sweden; c Center for HIV and STIs, National Institute for Communicable Diseases of the National Health Laboratory Service, Johannesburg, South Africa; d Centre for the AIDS Programme of Research in South Africa (CAPRISA), University of KwaZulu-Natal, Durban, South Africa; e Department of Public Health Medicine, School of Nursing and Public Health, University of KwaZulu-Natal, Durban, South Africa; f Department of Epidemiology, Columbia University, New York, New York, USA; g Medical Research Council Antibody Immunity Research Unit, University of Witwatersrand, Johannesburg, South Africa; h National Health Laboratory Services of South Africa, Johannesburg, South Africa; i Wellcome Centre for Infectious Disease Research in Africa, University of Cape Town, Observatory, South Africa; Emory University

**Keywords:** broadly neutralizing antibodies, coinfection, human immunodeficiency virus, neutralizing antibodies, vaccine cocktails, vaccines

## Abstract

Induction of broadly neutralizing antibodies (bNAbs) to HIV and other diverse pathogens will likely require the use of multiple immunogens. An understanding of the dynamics of antibody development to multiple diverse but related antigens would facilitate the rational design of immunization strategies. Here, we characterize, in detail, the development of neutralizing antibodies in three individuals coinfected with several divergent HIV variants. Two of these coinfected individuals developed additive or cross-neutralizing antibody responses. However, interference was observed in the third case, with neutralizing antibody responses to one viral variant arising to the near exclusion of neutralizing responses to the other. Longitudinal characterization of the diversity in the Envelope glycoprotein trimer (Env) structure showed that in the individual who developed the broadest neutralizing antibodies, circulating viruses shared a conserved epitope on the trimer apex that was targeted by cross-neutralizing antibodies. In contrast, in the other two individuals, diversity was distributed across Env. Taken together, these data highlight that multiple related immunogens can result in immune interference. However, they also suggest that immunogen cocktails presenting shared, conserved neutralizing epitopes in a variable background may focus broadly neutralizing antibody responses to these targets.

**IMPORTANCE** Despite being the focus of extensive research, we still do not know how to reproducibly elicit cross-neutralizing antibodies against variable pathogens by vaccination. Here, we characterize the antibody responses in people coinfected with more than one HIV variant, providing insights into how the use of antigen “cocktails” might affect the breadth of the elicited neutralizing antibody response and how the relatedness of the antigens may shape this.

## INTRODUCTION

Induction of broadly neutralizing HIV antibodies (bNAbs) will likely be necessary for an effective HIV vaccine. Recombinant, soluble, stabilized HIV envelope glycoprotein (Env) trimers that resemble the native trimer have been shown to elicit neutralizing antibodies in animal models (1). However, these antibodies have been largely strain-specific, and new strategies to broaden responses remain a priority. One approach to achieve this is the coadministration of multiple immunogen variants (vaccine “cocktails”). Such cocktails could drive multiple independent neutralizing antibody responses to each immunogen or potentially favor responses to conserved epitopes. However, multiple unrelated stabilized Env trimers, administered together as a cocktail or sequentially, failed to promote cross-neutralizing antibodies in rabbits or macaques (2, 3). For this approach, it is not clear how much diversity is required, nor how such immunogens should be administered in order to promote the development of bNAbs. While bNAbs have not been successfully elicited by immunization, they develop in a subset of HIV-infected individuals. Insights from studies of these individuals continue to shape the next generation of vaccine strategies that hope to elicit similar responses.

In the majority of cases, HIV infection is established by a single founder variant. However, in ~20 to 30% of cases, infection can be traced to more than one founder (multivariant transmission) (4, 5). In a subset of these cases, highly diverged Env variants are evident, likely representing independent transmission events from different donors or multivirus transmission from a donor that was infected by two or more phylogenetically distinct variants. Teasing apart the neutralizing antibody responses to multiple Env antigens in these cases could provide information on how vaccines might be designed to promote cross-neutralization.

We previously characterized the antibody responses in five HIV-superinfected individuals and found that with sequential exposure to two HIV variants, the second variant failed to efficiently recruit cross-neutralizing memory B cells specific for conserved epitopes (6). Here, we expand this investigation to determine whether coexposure to two HIV variants (coinfection) rather than sequential exposure may better favor the development of HIV cross-neutralizing antibodies. We characterize, in-depth, the neutralizing antibody responses in three individuals infected with two or more diverse HIV variants prior to seroconversion (defined here as coinfection) and discuss the implications for the design of polyvalent immunogen cocktails.

## RESULTS

**FIG 1:**
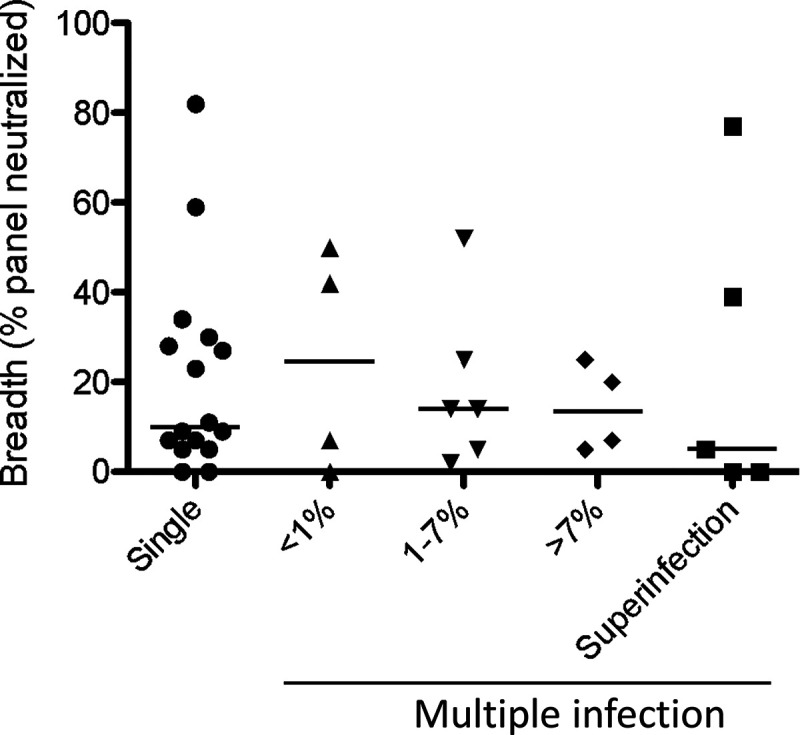
Neither multiplicity nor diversity of infection was associated with broader neutralizing antibody responses. Neutralization breadth at 3 years postinfection was previously quantified against a heterologous virus panel (*N* = 44) encompassing subtypes A, B, and C (14). “Single” indicates clinical infection consistent with a single transmitted/founder. “Superinfection” indicates infection following seroconversion with a second phylogenetically unlinked strain. Individuals coinfected with >1 variant prior to seroconversion are grouped according to the genetic distance in Env between the infecting variants.

We previously screened participants in the CAPRISA 002 acute infection cohort for cases of HIV dual infection, identifying 19 participants infected with multiple variants (7, 8), 14 of whom had evidence of multiple variants at the first seropositive visit. To determine whether coinfection with diverse variants generally led to broader neutralizing antibody responses, we assessed neutralization breadth at 2 and 3 years postinfection and compared this to participants likely infected with a single variant (*N* = 16). We found that coinfection alone was not sufficient to broaden responses compared to singly infected individuals (Fig. 1).

To define the immune response to multiple antigens, we next characterized the neutralizing antibody responses for three of the coinfected participants in whom single-genome sequencing of *env* identified coinfection with the divergent variants; each case represented a distinct antigenic exposure. We aimed to establish whether coinfection led to independent responses (additive) or promoted a response to epitopes conserved in both (immunofocusing) or whether the responses to distinct viruses competed with one another leading to lower titers than would have arisen following a monovalent exposure (interference). Further, we aimed to identify whether any antigenic features were associated with these outcomes.

To characterize the neutralizing antibody response to multiple variants in each of the three participants, we molecularly cloned representative Envelopes (Envs) from samples taken at the first HIV-positive visit and at approximately 3, 6, and 12 months postinfection. We generated Env-pseudotyped viruses and evaluated the titers and kinetics of the plasma neutralizing antibody response to each Env clone using longitudinal plasma samples from the first 2 years of infection. These are described below.

### Coinfection resulting in antibody interference (CAP267).

**FIG 2:**
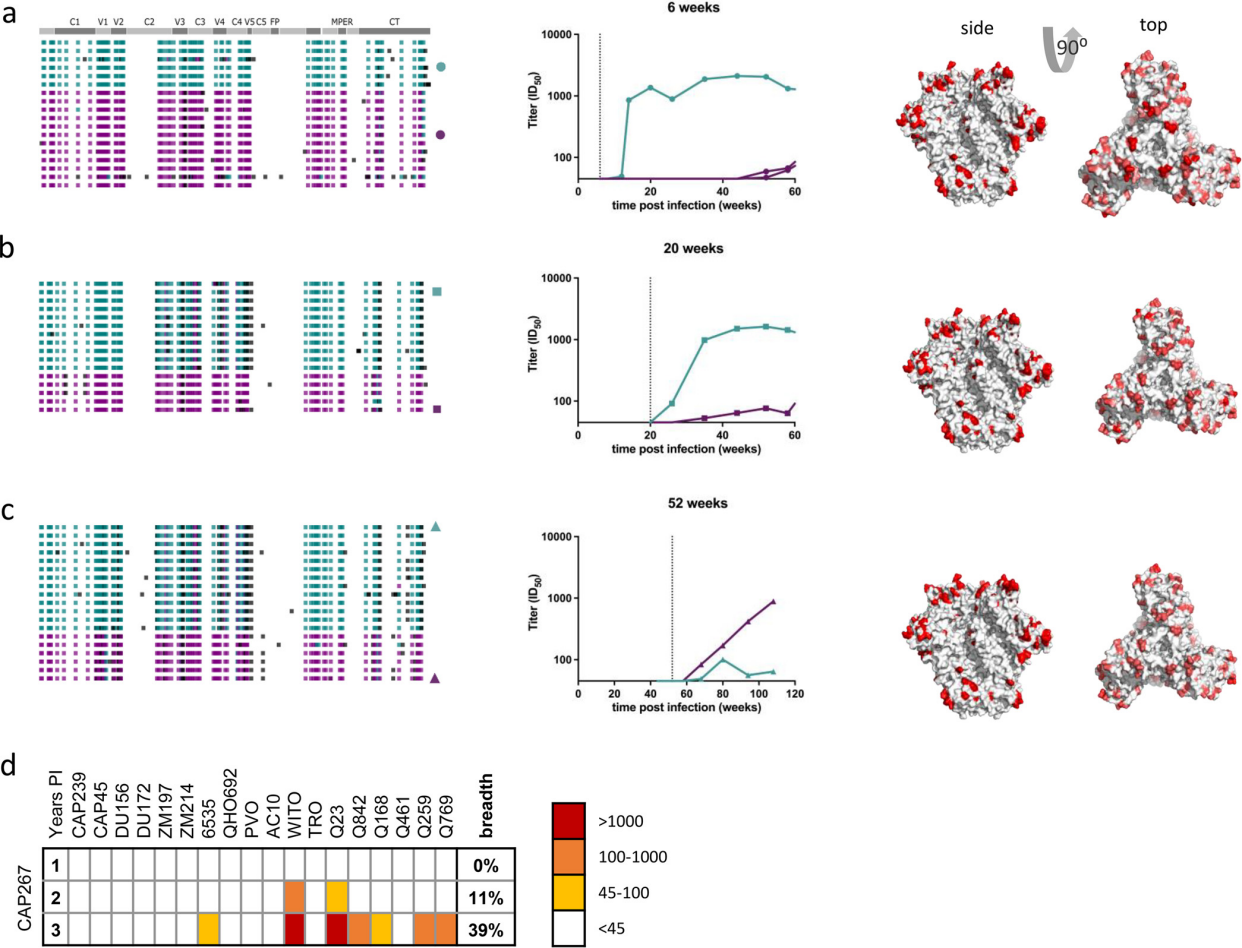
Interference in the autologous neutralizing antibody response in CAP267. CAP267 was coinfected with variants harboring 12% amino acid diversity uniformly distributed across Env. (a to c) The left panels show sequence highlighter plots summarizing the *env* sequences from 1 month (a), 6 months (b), and 12 months (c) postinfection, where each row represents a single-genome sequence. Teal and purple ticks represent nucleotides that match those in the two major variants (predominating transmitted/founder variants), respectively. White ticks represent sites harboring a nucleotide conserved in both variants, and black ticks represent polymorphisms matching neither major variant. MPER, membrane-proximal external region; FP, fusion peptide; CT, cytoplasmic tail. The middle panels depict longitudinal, autologous neutralization of representatives from both major variants cloned from the corresponding plasma sample. The vertical dashed lines identify the time point from which the Env clones were isolated. Potent neutralization of one variant with weak or undetectable neutralization of the other was evident at all three time points. The right panels depict diversity overlaid onto the Env structure (PDB code 4ZMJ), where white represents conservation, and redder colors denote increasing diversity at that site. (d) Neutralization of 18 heterologous viruses at 1 to 3 years postinfection, where white represents no detectable cross-neutralization (ID_50_ < 45), and warmer colors represent more potent neutralization, as shown in the key. Years PI, years postinfection.

Shortly following infection, CAP267 harbored two distinct but recombined viral populations that differed by up to 12% in Env. The recombination can readily be visualized in the highlighter plot (Fig. 2a), in that the large regions of white indicate that these otherwise diverse variants were identical over these subgenomic regions. Both variants were detectable at every sampled time point thereafter, and they remained distinct with no significant further recombination in Env evident over the first year of infection (Fig. 2a to c, left panels). Overlaying the locations of amino acid differences between these lineages onto the quaternary Env structure (PDB code 4ZMJ) (9) revealed that these differences were uniformly distributed across the surface of the trimer (Fig. 2a to c, right panels).

Characterizing the longitudinal, autologous neutralizing antibody response to representative variants sampled at each time point revealed preferential neutralization of one variant, with titers to the second consistently remaining low (<100) or below the limit of detection (<45) (Fig. 2a to c, middle panels). This preferential neutralization switched from one variant to the other after 1 year postinfection (Fig. 2c), indicating that the differential neutralization was not due to inherently low immunogenicity of one variant. The weak nAb titers that were evident were significantly lower than the cohort average and represented some of the lowest titers observed (Fig. S1), suggesting immune interference.

Interestingly, the preferentially targeted variant frequently represented the minority population (as determined from the frequency in single-genome amplification [SGA] sequences) at the time that nAb responses arose, highlighting that antigen load is not the dominant driver of immunogenicity/immunodominance. CAP267 went on to develop some cross-neutralizing activity with plasma samples from 2 and 3 years postinfection able to neutralize 11 and 39% of heterologous viruses, respectively (Fig. 2d). However, even after the development of cross-neutralizing activity, autologous neutralizing responses to the dominant variant at 12 months postinfection remained barely detectable (Fig. 2c). This indicates that the cross-neutralizing antibodies failed to target epitopes common to both autologous variants.

### Coinfection resulting in additive antibody responses (CAP137).

**FIG 3:**
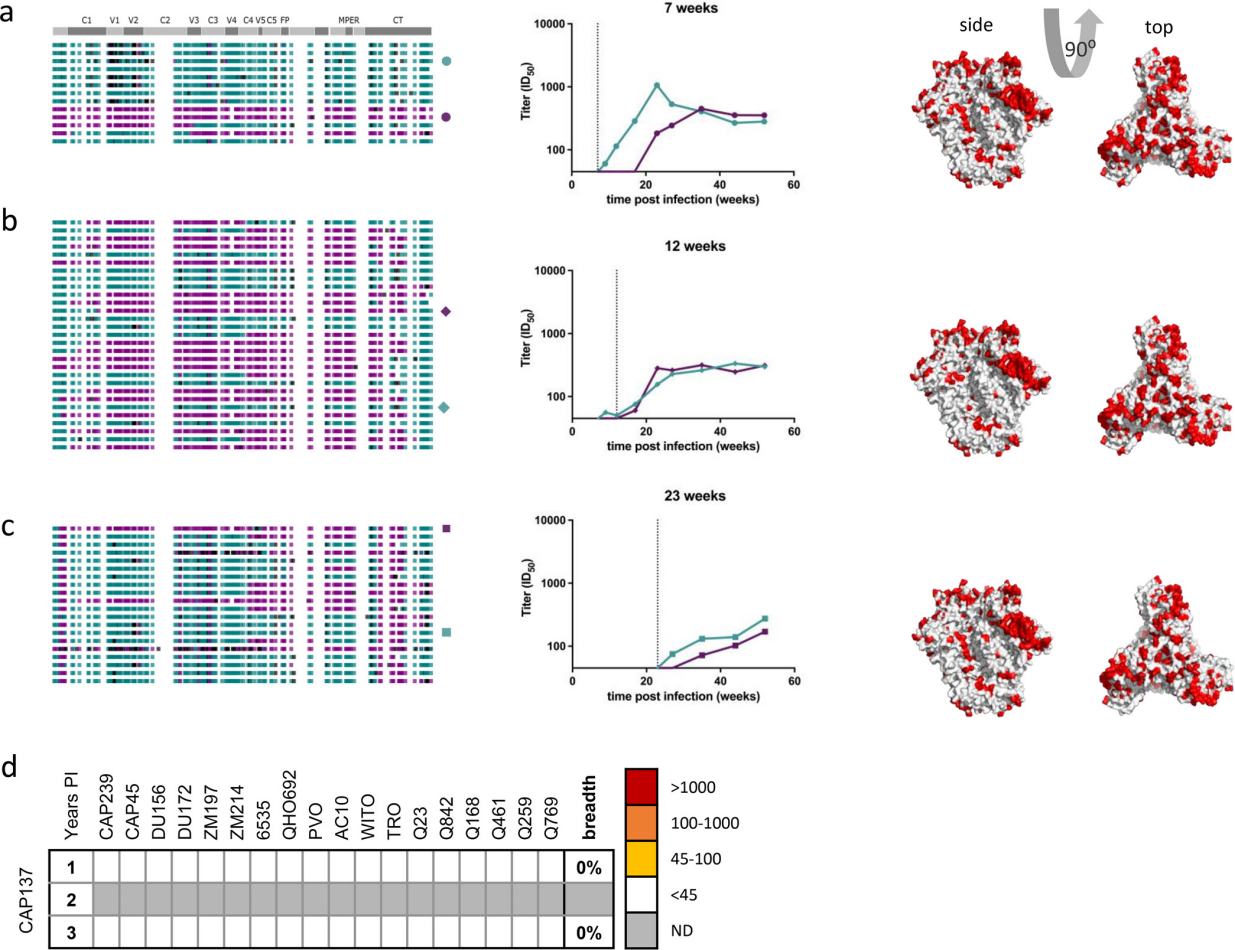
Autologous and heterologous neutralizing antibody responses in CAP137. CAP137 was coinfected with unlinked variants displaying 21% amino acid diversity distributed across Env. (a to c) The left panels depict sequence highlighter plots summarizing *env* single-genome sequences sampled in plasma from 1 month (a), 3 months (b), and 6 months (c) postinfection, where each row represents a single-genome sequence. Teal and purple ticks represent nucleotides that match those in the two major variants, respectively. White ticks represent sites harboring a nucleotide conserved in both variants, and black ticks represent polymorphisms matching neither major variant. MPER, membrane-proximal external region; FP, fusion peptide; CT, cytoplasmic tail. The middle panels depict longitudinal, autologous neutralization of representatives from both major variants in the corresponding plasma sample. The vertical dashed lines identify the time point from which the Env clones were isolated. The right panels depict diversity overlaid onto the Env structure (PDB code 4ZMJ), where white represents conservation, and redder colors denote increasing diversity (Entropy) at that site. (d) CAP137 plasma from 1 and 3 years postinfection (PI) was unable to cross-neutralize any of 18 heterologous viruses tested. Plasma from 2 years PI was not tested. ND, not done.

Donor CAP137 was infected with two highly divergent, phylogenetically unlinked variants differing by ~21% in Env. Amino acid differences between the two variants were uniformly distributed over the surface of the Env trimer (Fig. 3a, right panel). Both variants were observed at 3 and 6 months (12-month samples were unavailable), although they harbored subgenomic fragments inherited by recombination between cotransmitted variants (Fig. 3a to c, left panels). While neutralizing antibody responses arose to both variants (Fig. 3a to c, middle panels), responses to one variant were relatively delayed and first detectable only ~6 months postinfection (8 to 14 weeks after the nAb response to the first variant), which is suggestive of some degree of immune interference in the response to this early clone (Fig. S2). However, representative Envs of both variants sampled at 3 and 6 months postinfection were neutralized with similar kinetics and similarly low titers (Fig. 3b and c, middle panels): ID_50_ titers peaked at ~350 for 3-month Envs and ~275 for 6-month Envs. Despite neutralization of both autologous Env variants, no cross-neutralization of any heterologous viruses was detectable by 3 years postinfection (Fig. 3d). This indicates that high levels of diversity dispersed across cocirculating Envs resulted in separate responses targeting nonconserved epitopes on each variant.

### Coinfection resulting in cross-neutralizing responses (CAP37).

**FIG 4:**
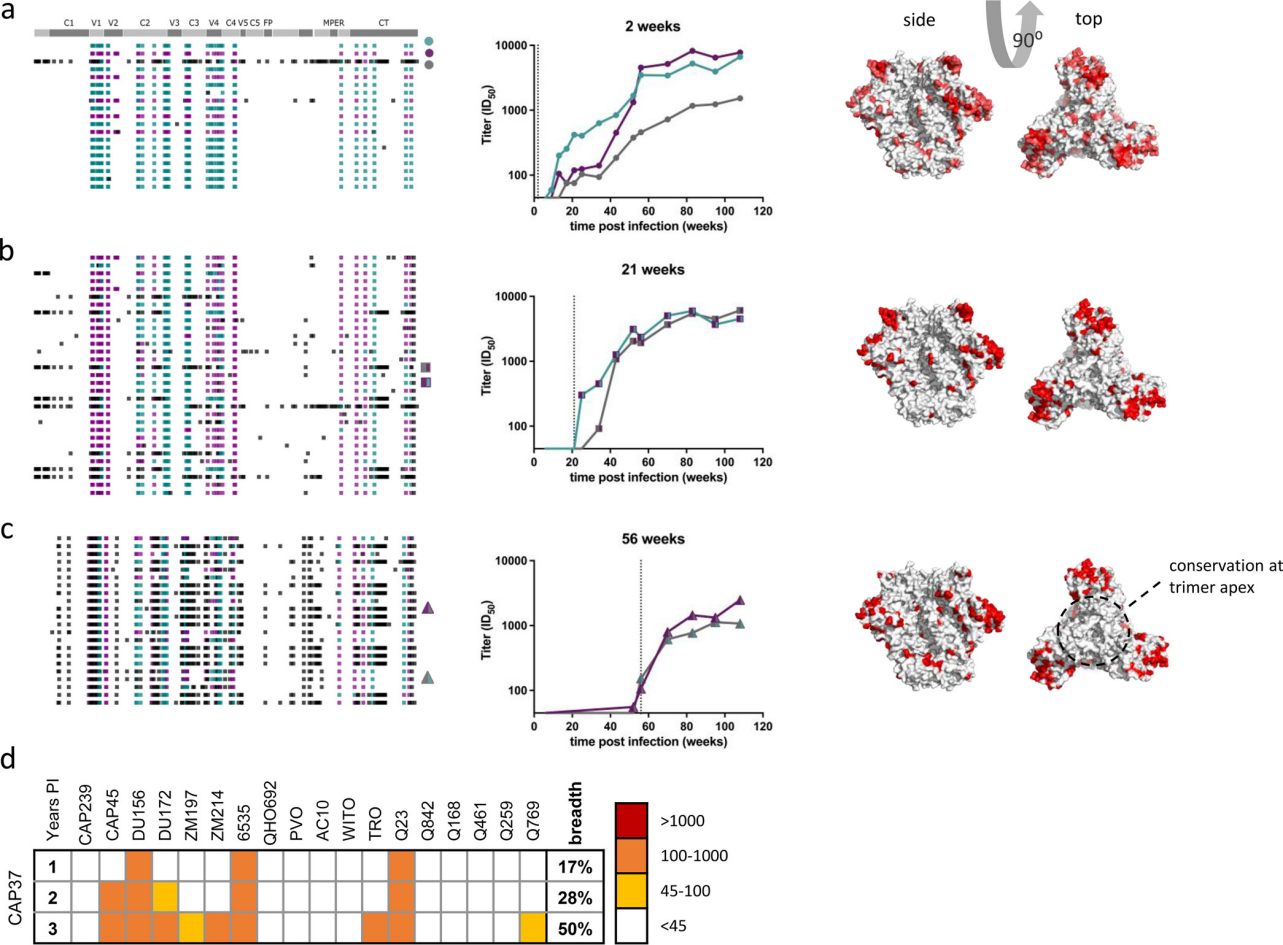
Potent neutralization of variants in CAP37. CAP37 was coinfected with at least three recombinant variants harboring up to 16% amino acid diversity, clustered in Env. (a to c) The left panels depict sequence highlighter plots summarizing the *env* sequences at 2 weeks (a), 21 weeks (b), and 46 months (c) postinfection, where each row represents a single-genome sequence. Teal and purple ticks represent nucleotides that match those in the two major variants, respectively. White ticks represent sites harboring a nucleotide conserved in both variants, and black ticks represent polymorphisms matching neither major variant. MPER, membrane-proximal external region; FP, fusion peptide; CT, cytoplasmic tail. The middle panels depict potent autologous neutralization of representative variants cloned from the corresponding plasma sample. The vertical dashed lines identify the time point from which the Env clones were isolated. The right panels depict diversity overlaid onto the Env structure (PDB code 4ZMJ), where white represents conservation, and redder colors denote increasing diversity at that site. (d) CAP37 developed cross-neutralizing activity against a panel of 18 viruses, with plasma sampled 3 years postinfection (PI) capable of neutralizing 50% of heterologous viruses tested.

CAP37 was coinfected with at least three variants. Two of the variants differed by only 6% in Env, while the third was highly diverged, differing by ~16% in Env. However, all three variants shared subgenomic regions of high homology within Env, likely inherited through recombination (Fig. 4a to c, left panels). Shared regions across these variants included known bNAb targets, including V2 at the Env trimer apex, the membrane-proximal external region (MPER), and the fusion peptide (Fig. 3a, right panel). Locations of high diversity mapped to highly immunogenic regions (including C3V4) (10, 11) and to regions that are more accessible (Fig. S3).

The kinetics and titer profile of nAb responses to these early Env clones suggest that responses were at least additive (Fig. 4a). All three of the transmitted variants were relatively potently neutralized with ID_50_ titers peaking between 2,000 and 10,000. Titers and kinetics of neutralization were similar for the dominant circulating 6- and 12-month Envs (Fig. 4b and c), suggestive of a single response capable of cross-neutralizing both variants (although quantitatively similar but independent responses to both variants cannot be excluded). This coincided in time with further reduced diversity at the trimer apex, likely through recombination, despite high diversity elsewhere (Fig. 4b and c, right panels).

CAP37 developed cross-neutralizing activity, neutralizing 28 and 50% of a heterologous virus panel at 2 and 3 years postinfection, respectively (Fig. 4d). As the trimer apex including V2 is a frequent target of broadly neutralizing antibodies (12–14), we sought to determine whether cross-neutralization in CAP37 was due to antibodies targeting this conserved region. Dependency on the *N*-linked glycan at position 160 and a lysine at position 169 is common to many broadly neutralizing antibodies specific for V2 (15, 16). Therefore, we introduced mutations at these sites into three heterologous pseudoviruses neutralized by CAP37 and evaluated their effect on neutralization by plasma sampled between 18 and 24 months postinfection. N160A and K169E mutations substantially reduced neutralization of heterologous viruses, ConC, and CAP45 by the 2-year plasma sample (by between 7- and 29-fold; Table S1), indicating that these viruses were predominantly neutralized by V2-directed antibodies. Neutralization of Du156 by plasma at approximately 1.5 years postinfection was moderately (~4-fold) reduced by the introduction of an N160K (Table S1). However, at 2 years postinfection, the same mutation enhanced neutralization, suggesting that the removal of the *N*-linked glycan may have better exposed the targeted epitope. However, the K169E mutation did not significantly affect neutralization of Du156. Immune pressure from neutralizing antibodies typically drives sequence evolution, and evidence of strong diversifying selection in longitudinal V2 sequences was apparent. However, pressure was not centered on the C strand of V2 (the target of all well-characterized V2 broadly neutralizing antibodies) but adjacent in the V2 loop. Taken together, these data suggest that apex-targeting antibodies develop in CAP37 and can mediate cross-neutralization, although they do not completely account for the plasma breadth.

## DISCUSSION

Designing a vaccine able to elicit broadly neutralizing antibodies against HIV has proven to be an immense scientific challenge. Multivalent HIV envelope glycoprotein (Env) cocktails have successfully elicited neutralizing antibodies in animal models but to date have been largely unsuccessful at eliciting any substantial neutralizing antibody breadth. Species-specific differences in the antibody response to Env immunogens may nevertheless hinder their translation to human vaccine regimens. An understanding of the dynamics of antibody responses to multiple HIV Env antigens in humans would facilitate the design of vaccine cocktails. We therefore characterized the neutralizing antibody response over time in three HIV coinfected individuals, encompassing immunological exposure to: (i) two diverse, unlinked Envs, (ii) two related Envs with diversity spread over the trimer, and (iii) two diverse Envs that had recombined such that large regions were conserved in both variants. We find that coinfection alone was not sufficient to broaden neutralizing antibody responses. Furthermore, in the three cases of coinfection studied in detail, various features of antigen diversity led to markedly different outcomes. Two diverse Envs led to additive antibody responses, while profound interference followed coinfection with more similar Envs. Importantly, coexposure to related Envs that presented shared conserved regions but maintained high diversity in exposed, immunodominant regions was associated with increased breadth.

In the case of exposure to two diverse, unlinked subtype C Envs differing by 21% (CAP137), titers and kinetics were indicative of independent neutralizing antibody responses to each variant. Furthermore, responses to one of the early clones in CAP137 were significantly delayed, and titers to both variants following the targeting of the second variant were low. This is consistent with the responses seen in animals immunized with multivalent cocktails of Envs. Immunization with two stabilized trimers elicited distinct neutralizing antibody lineages to each variant even though both shared immunodominant “glycan holes” (17). Similarly, polyvalent immunization with cocktails of stabilized HIV Env trimers from multiple subtypes generally elicited neutralizing antibody responses to each of the Envs, but no significant broadening of the responses to Envs not included in the immunization was evident (2, 3). These multivalent stabilized Env trimer formulations also elicited 3- to 7-fold lower titers compared to corresponding monovalent immunizations. Together with our observations here, these results indicate that cocktails of diverse Envs, derived from sequences from unlinked infections, are unlikely to elicit cross-neutralizing responses and may be subject to competition.

In contrast to CAP137, participant CAP267 was coinfected with two more closely related Envs (12%), with differences dispersed across the trimer. This allowed us to investigate whether exposure to more similar trimers may have promoted cross-neutralizing responses. However, the antibody response in CAP267 displayed a profound immunodominance, neutralizing one virus to the near exclusion of nAb responses to the other. This suggests that the use of similar antigens may be subject to more pronounced interference when many presented B-cell epitopes are not well conserved. More similar antigens are more likely to share CD4 helper epitopes, and competition between independent B-cell lineages for the same CD4 helper population would drive this interference (18). The neutralizing antibody response in CAP267 also preferentially targeted minority variants throughout the first 2 years of infection, suggesting that B-cell receptor (BCR) affinity was a more prominent driver of specificity than antigen availability.

Interestingly, potent neutralization of two variants was evident in a third participant, CAP37, coinfected with two similarly divergent Envs (16% different in Env). Unlike the previous two participants, in whom diversity was dispersed across Env, in CAP37, the diversity was highly localized, with large regions, including V2 at the trimer apex, conserved in both variants through recombination. Furthermore, this participant developed the broadest neutralizing antibody response, and cross-neutralization was mediated, in part, by neutralizing antibodies targeting this conserved region. While these data indicate that coexposure to two diverse Envs harboring the same trimer apex may have promoted this response to a conserved epitope, this is also a relatively common target of neutralization in singly infected individuals (11, 19–21).

In summary (Fig. S4), administering multiple diverse Envs in a cocktail may nominally improve breadth by driving multiple, nearly additive responses, but we suggest they are unlikely to be sufficient to drive broadly neutralizing antibody lineages. Further, we show here that neutralizing antibody responses to multiple, similar Envs can be subject to interference, where nAb responses are elicited to one Env at the expense of responses to the other. Importantly, when diversity was clustered in immunogenic regions but more conserved regions were preserved, neutralizing antibodies may have been directed toward these conserved epitopes. While these observations are based on only a few individuals, these data are consistent with and extend preclinical data from the immunization of animals with immunogen cocktails (2, 3). Taken together, they suggest that the coadministration of multiple stabilized Env trimers with diversity introduced in key regions, along with parallel strategies to reduce the immunodominance of strain-specific epitopes, may represent one path to a cross-neutralizing antibody response.

## MATERIALS AND METHODS

### Human subjects.

Samples were provided from participants from the CAPRISA 002 Acute Infection Study established in 2004 (22). This cohort recruited high-risk, recently HIV-infected women from Durban and Vulindlela, KwaZulu-Natal, South Africa, as well as from the CAPRISA 004 study (23). The timing of infection was estimated as either the midpoint between the last antibody-negative and first antibody-positive visits or 14 days prior to an RNA-positive, antibody-negative sample. HIV-positive participants were followed longitudinally, and plasma samples were taken weekly for 3 weeks, fortnightly until approximately 3 months postinfection, monthly until approximately 1 year postinfection, and quarterly thereafter. Plasma was stored in either EDTA or acid citrate dextrose (ACD) to prevent coagulation and stored at −80°C until use. Participants in this study were antiretroviral therapy (ART) naive and were initiated on ART consistent with the prevailing South African ART guidelines. Ethical approval for this study was received from the ethics committees of the University of Cape Town (025/2004), the University of KwaZulu-Natal (E013/04), and the University of the Witwatersrand (MM040202), and all participants provided written, informed consent.

### Cell lines.

TZM-bl (JC53-bl) cells, engineered by J. Kappes and X. Wu, were obtained from the National Institutes of Health (NIH) AIDS Research and Reference Reagent Program (catalog no. 8129). HEK293T cells were obtained from George Shaw (University of Alabama, Birmingham, AL). Both cell lines were maintained in Dulbecco’s modified Eagle’s medium (Gibco, Life Technologies, Carlsbad, CA), containing 4.5 g/liter glucose, l-glutamine, sodium pyruvate, and supplemented with 1% penicillin and streptomycin, 25 mM HEPES (Sigma-Aldrich, St. Louis, MO), and 10% heat-inactivated fetal bovine serum (FBS) (Biochrom, Cambridge, UK). The cells were cultured at 37°C in a humidified incubator with 5% CO_2_, and the monolayers were disrupted when nearing confluence with trypsin-EDTA.

### Single-genome sequencing.

Plasma viral RNA was extracted from 200 μL or 400 μL of plasma using either the Roche MagNApure (Roche Applied Science, Mannheim, Germany) or QIAamp viral RNA minikits (Qiagen, Valencia, CA). RNA was reversed-transcribed to cDNA using Superscript III (Invitrogen, Life Technologies, Carlsbad, CA) as per the manufacturer’s instructions. *Env* cassettes were amplified from the cDNA in a nested PCR by SGA using 0.025 units of Platinum *Taq* High Fidelity (Invitrogen, Life Technologies, Carlsbad, CA) per 20-μL reaction, as previously described (4, 5). The primers used in the outer reaction were 5′-GGGTTTATTACAGGGACAGCAGAG-3′ (HXB2 nucleotides [nt] 4900 to 4923) and 5′-GCACTCAAGGCAAGCTTTATTGAGGCTTA-3′ (HXB2 nt 9604 to 9632). The inner primers used were 5′-CACC GGCTTAGGCATCTCCTATAGCAGGAAGAA-3′ (HXB2 nt 5954 to 5982) and 5′-TTGCCAATCAAGGAAGTAGCCTTGTGT-3′ (HXB2 nt 9145 to 9171). The outer reaction thermal cycling conditions were as follows: initial denaturation at 94°C for 2 min; followed by 35 cycles of 94°C for 15 s, 55°C for 30 s, and 68°C for 4 min; followed by a final extension for 1 cycle at 68°C for 20 min. The inner reaction thermal cycling conditions were the same as above, for 45 cycles. Amplicons were directly sequenced using an ABI3000 genetic analyzer and BigDye Terminator reagents (Applied Biosystems, Foster City, CA) using 12 primers, by the Central Analytic Facility at the University of Stellenbosch, South Africa. Contigs were assembled using Sequencher 4.10.1 (Gene Codes, Ann Arbor, MI). All sequences were screened for contamination against a database of all sequences generated in the laboratory, including all constructs used.

### Molecular cloning.

Amplicons were ligated into pcDNA3.1-Topo Directional Cloning Vector (Invitrogen, Life Technologies, Carlsbad, CA), transformed into Top10 chemically competent Escherichia coli cells (Invitrogen, Life Technologies, Carlsbad, CA) as per the manufacturer’s instructions, and cultured on Luria-Bertani agar supplemented with 100 μg/mL carbenicillin (Sigma-Aldrich, St. Louis, MO). Plasmid purification was performed using the QIAprep spin miniprep, or the QIAfilter plasmid midi kits (Qiagen, Valencia, CA), as per the manufacturer’s instructions. Plasmids were sequenced as described above in order to ensure the clone contained no nonsynonymous mutations relative to the SGA-derived *env* sequence.

### Neutralization assays.

Env-pseudotyped viruses were generated by cotransfecting *env* plasmids with pSG3ΔEnv at a 1:2 ratio into HEK293T cells using FuGENE 6 (Applied Science, Indianapolis, IA) or PolyFect (Qiagen, Valencia, CA) per the manufacturer’s instructions. Pseudoviruses were harvested from the supernatant 48 h following transfection, filtered through a 0.45-μm filter (Millipore, Merck, Billerica, MA), supplemented up to 20% FBS, and stored at −80°C until use. Neutralization assays were performed as described previously (14). Assays were performed in duplicate wells and repeated at least twice using aliquots of the same plasma draw. Neutralization breadth for the cohort was quantified as the proportion of heterologous viruses from a multisubtype pseudovirus panel of 44 pseudotyped heterologous viruses each plasma sample was able to neutralize at ID_50_ titers >45 (14). Breadths at 1, 2, and 3 years were also estimated for coinfected participants using a reduced panel of 18 heterologous viruses.

### Mutagenesis.

Point mutants were generated using the QuikChange Lightning site-directed mutagenesis kit (Agilent Technologies, Santa Clara, CA) as per the manufacturer’s instructions.

### Visualization of Env diversity.

To visualize the dispersion in diversity, we generated Env models colored by variability, where the per site variability was calculated as the Entropy (nats) at each site in the alignment of single-genome amino acid sequences, sampled at each time point from that individual. An average of 80 (range, 68 to 91) single-genome sequences were analyzed for each individual in total. We then replaced the B-factor values for each position in the Env structural model (PDB code 4ZMJ) with the corresponding entropy score and colored the model according to these values in PyMOL, scaling from white (lowest) to red (highest). Any insertions in the structural model relative to query alignments are shown as 0.0 (white) by default.

### Characterizing selection pressure.

Positive selection in alignments was assessed using MEME (24) and FUBAR (25) on the datamonkey web server (www.datamonkey.org). Alignments were partitioned to account for the confounding effects of recombination (26) using GARD (27).

### Diffusion accessibility.

Diffusion accessibility was estimated using the UCLA webtool (http://services.mbi.ucla.edu/DiffAcc/) (28) with PDB accession code 4ZMJ (9).

### Code availability.

The Julia code used to generate the sequence highlighter plots is available at https://github.com/MurrellGroup/highlighterplotting with no restrictions for use.

### Data availability.

Further information and requests for resources and reagents should be directed to and will be fulfilled by the lead contact, Carolyn Williamson (Carolyn.Williamson@uct.ac.za).

## References

[1] Sanders RW, Moore JP. 2017. Native-like Env trimers as a platform for HIV-1 vaccine design. Immunol Rev 275:161–182. 10.1111/imr.12481. 28133806 PMC5299501

[2] Klasse PJ, LaBranche CC, Ketas TJ, Ozorowski G, Cupo A, Pugach P, Ringe RP, Golabek M, van Gils MJ, Guttman M, Lee KK, Wilson IA, Butera ST, Ward AB, Montefiori DC, Sanders RW, Moore JP. 2016. Sequential and simultaneous immunization of rabbits with HIV-1 envelope glycoprotein SOSIP.664 trimers from clades A, B and C. PLoS Pathog 12:e1005864. 10.1371/journal.ppat.1005864. 27627672 PMC5023125

[3] de la Peña AT, de Taeye SW, Sliepen K, LaBranche CC, Burger JA, Schermer EE, Montefiori DC, Moore JP, Klasse PJ, Sanders RW. 2018. Immunogenicity in rabbits of HIV-1 SOSIP trimers from clades A, B, and C, given individually, sequentially, or in combination. J Virol 92:e01957-17. 29367243 10.1128/JVI.01957-17 PMC5874403

[4] Keele BF, Giorgi EE, Salazar-Gonzalez JF, Decker JM, Pham KT, Salazar MG, Sun C, Grayson T, Wang S, Li H, Wei X, Jiang C, Kirchherr JL, Gao F, Anderson JA, Ping L-H, Swanstrom R, Tomaras GD, Blattner WA, Goepfert PA, Kilby JM, Saag MS, Delwart EL, Busch MP, Cohen MS, Montefiori DC, Haynes BF, Gaschen B, Athreya GS, Lee HY, Wood N, Seoighe C, Perelson AS, Bhattacharya T, Korber BT, Hahn BH, Shaw GM. 2008. Identification and characterization of transmitted and early founder virus envelopes in primary HIV-1 infection. Proc Natl Acad Sci USA 105:7552–7557. 10.1073/pnas.0802203105. 18490657 PMC2387184

[5] Abrahams M-R, Anderson JA, Giorgi EE, Seoighe C, Mlisana K, Ping L-H, Athreya GS, Treurnicht FK, Keele BF, Wood N, Salazar-Gonzalez JF, Bhattacharya T, Chu H, Hoffman I, Galvin S, Mapanje C, Kazembe P, Thebus R, Fiscus S, Hide W, Cohen MS, Karim SA, Haynes BF, Shaw GM, Hahn BH, Korber BT, Swanstrom R, Williamson C, CAPRISA Acute Infection Study Team, Center for HIV-AIDS Vaccine Immunology Consortium. 2009. Quantitating the multiplicity of infection with human immunodeficiency virus type 1 subtype C reveals a non-Poisson distribution of transmitted variants. J Virol 83:3556–3567. 10.1128/JVI.02132-08. 19193811 PMC2663249

[6] Sheward DJ, Marais J, Bekker V, Murrell B, Eren K, Bhiman JN, Nonyane M, Garrett N, Woodman ZL, Abdool Karim Q, Abdool Karim SS, Morris L, Moore PL, Williamson C. 2018. HIV superinfection drives *de novo* antibody responses and not neutralization breadth. Cell Host Microbe 24:593–599. 10.1016/j.chom.2018.09.001. 30269971 PMC6185870

[7] Woodman Z, Mlisana K, Treurnicht F, Abrahams M-R, Thebus R, Karim SA, Williamson C, Caprisa Acute Infection Study Team. 2011. Short communication decreased incidence of dual infections in South African subtype C-infected women compared to a cohort ten years earlier. AIDS Res Hum Retroviruses 27:1167–1172. 10.1089/aid.2010.0162. 21198409 PMC3206740

[8] Redd AD, Mullis CE, Wendel SK, Sheward D, Martens C, Bruno D, Werner L, Garrett NJ, Abdool Karim Q, Williamson C, Porcella SF, Quinn TC, Abdool Karim SS. 2014. Limited HIV-1 superinfection in seroconverters from the CAPRISA 004 Microbicide Trial. J Clin Microbiol 52:844–848. 10.1128/JCM.03143-13. 24371237 PMC3957790

[9] Kwon YD, Pancera M, Acharya P, Georgiev IS, Crooks ET, Gorman J, Joyce MG, Guttman M, Ma X, Narpala S, Soto C, Terry DS, Yang Y, Zhou T, Ahlsen G, Bailer RT, Chambers M, Chuang G-Y, Doria-Rose NA, Druz A, Hallen MA, Harned A, Kirys T, Louder MK, O’Dell S, Ofek G, Osawa K, Prabhakaran M, Sastry M, Stewart-Jones GBE, Stuckey J, Thomas PV, Tittley T, Williams C, Zhang B, Zhao H, Zhou Z, Donald BR, Lee LK, Zolla-Pazner S, Baxa U, Schön A, Freire E, Shapiro L, Lee KK, Arthos J, Munro JB, Blanchard SC, Mothes W, Binley JM, et al. 2015. Crystal structure, conformational fixation and entry-related interactions of mature ligand-free HIV-1 Env. Nat Struct Mol Biol 22:522–531. 10.1038/nsmb.3051. 26098315 PMC4706170

[10] Moore PL, Gray ES, Morris L. 2009. Specificity of the autologous neutralizing antibody response. Curr Opin HIV AIDS 4:358–363. 10.1097/COH.0b013e32832ea7e8. 20048698 PMC3004050

[11] Moore PL, Gray ES, Choge IA, Ranchobe N, Mlisana K, Abdool Karim SS, Williamson C, Morris L, CAPRISA 002 Study Team. 2008. The c3-v4 region is a major target of autologous neutralizing antibodies in human immunodeficiency virus type 1 subtype C infection. J Virol 82:1860–1869. 10.1128/JVI.02187-07. 18057243 PMC2258729

[12] Landais E, Huang X, Havenar-Daughton C, Murrell B, Price MA, Wickramasinghe L, Ramos A, Bian CB, Simek M, Allen S, Karita E, Kilembe W, Lakhi S, Inambao M, Kamali A, Sanders EJ, Anzala O, Edward V, Bekker L-G, Tang J, Gilmour J, Kosakovsky-Pond SL, Phung P, Wrin T, Crotty S, Godzik A, Poignard P. 2016. Broadly neutralizing antibody responses in a large longitudinal sub-Saharan HIV primary infection cohort. PLoS Pathog 12:e1005369. 10.1371/journal.ppat.1005369. 26766578 PMC4713061

[13] Walker LM, Simek MD, Priddy F, Gach JS, Wagner D, Zwick MB, Phogat SK, Poignard P, Burton DR. 2010. A limited number of antibody specificities mediate broad and potent serum neutralization in selected HIV-1 infected individuals. PLoS Pathog 6:e1001028. 10.1371/journal.ppat.1001028. 20700449 PMC2916884

[14] Gray ES, Moore PL, Choge IA, Decker JM, Bibollet-Ruche F, Li H, Leseka N, Treurnicht F, Mlisana K, Shaw GM, Karim SSA, Williamson C, Morris L, CAPRISA 002 Study Team. 2007. Neutralizing antibody responses in acute human immunodeficiency virus type 1 subtype C infection. J Virol 81:6187–6196. 10.1128/JVI.00239-07. 17409164 PMC1900112

[15] Andrabi R, Voss JE, Liang C-H, Briney B, McCoy LE, Wu C-Y, Wong C-H, Poignard P, Burton DR. 2015. Identification of common features in prototype broadly neutralizing antibodies to HIV envelope V2 apex to facilitate vaccine design. Immunity 43:959–973. 10.1016/j.immuni.2015.10.014. 26588781 PMC4654981

[16] Gorman J, Soto C, Yang MM, Davenport TM, Guttman M, Bailer RT, Chambers M, Chuang G-Y, DeKosky BJ, Doria-Rose NA, Druz A, Ernandes MJ, Georgiev IS, Jarosinski MC, Joyce MG, Lemmin TM, Leung S, Louder MK, McDaniel JR, Narpala S, Pancera M, Stuckey J, Wu X, Yang Y, Zhang B, Zhou T, Mullikin JC, Baxa U, Georgiou G, McDermott AB, Bonsignori M, Haynes BF, Moore PL, Morris L, Lee KK, Shapiro L, Mascola JR, Kwong PD, NISC Comparative Sequencing Program. 2016. Structures of HIV-1 Env V1V2 with broadly neutralizing antibodies reveal commonalities that enable vaccine design. Nat Struct Mol Biol 23:81–90. 10.1038/nsmb.3144. 26689967 PMC4833398

[17] Yang YR, McCoy LE, van Gils MJ, Andrabi R, Turner HL, Yuan M, Cottrell CA, Ozorowski G, Voss J, Pauthner M, Polveroni TM, Messmer T, Wilson IA, Sanders RW, Burton DR, Ward AB. 2020. Autologous antibody responses to an HIV envelope glycan hole are not easily broadened in rabbits. J Virol 94:e01861-19. 10.1128/JVI.01861-19. 31941772 PMC7081899

[18] Victora GD, Schwickert TA, Fooksman DR, Kamphorst AO, Meyer-Hermann M, Dustin ML, Nussenzweig MC. 2010. Germinal center dynamics revealed by multiphoton microscopy with a photoactivatable fluorescent reporter. Cell 143:592–605. 10.1016/j.cell.2010.10.032. 21074050 PMC3035939

[19] Sagar M, Wu X, Lee S, Overbaugh J. 2006. Human immunodeficiency virus type 1 V1-V2 envelope loop sequences expand and add glycosylation sites over the course of infection, and these modifications affect antibody neutralization sensitivity. J Virol 80:9586–9598. 10.1128/JVI.00141-06. 16973562 PMC1617272

[20] Rong R, Li B, Lynch RM, Haaland RE, Murphy MK, Mulenga J, Allen SA, Pinter A, Shaw GM, Hunter E, Robinson JE, Gnanakaran S, Derdeyn CA. 2009. Escape from autologous neutralizing antibodies in acute/early subtype C HIV-1 infection requires multiple pathways. PLoS Pathog 5:e1000594. 10.1371/journal.ppat.1000594. 19763269 PMC2741593

[21] Moore PL, Ranchobe N, Lambson BE, Gray ES, Cave E, Abrahams M-R, Bandawe G, Mlisana K, Abdool Karim SS, Williamson C, Morris L, CAPRISA 002 Study. NIAID Center for HIV/AIDS Vaccine Immunology (CHAVI). 2009. Limited neutralizing antibody specificities drive neutralization escape in early HIV-1 subtype C infection. PLoS Pathog 5:e1000598. 10.1371/journal.ppat.1000598. 19763271 PMC2742164

[22] van Loggerenberg F, Mlisana K, Williamson C, Auld SC, Morris L, Gray CM, Abdool Karim Q, Grobler A, Barnabas N, Iriogbe I, Abdool Karim SS, CAPRISA 002 Acute Infection Study Team. 2008. Establishing a cohort at high risk of HIV infection in South Africa: challenges and experiences of the CAPRISA 002 acute infection study. PLoS One 3:e1954. 10.1371/journal.pone.0001954. 18414658 PMC2278382

[23] Abdool Karim Q, Abdool Karim SS, Frohlich JA, Grobler AC, Baxter C, Mansoor LE, Kharsany ABM, Sibeko S, Mlisana KP, Omar Z, Gengiah TN, Maarschalk S, Arulappan N, Mlotshwa M, Morris L, Taylor D, CAPRISA 004 Trial Group. 2010. Effectiveness and safety of tenofovir gel, an antiretroviral microbicide, for the prevention of HIV infection in women. Science 329:1168–1174. 10.1126/science.1193748. 20643915 PMC3001187

[24] Murrell B, Wertheim JO, Moola S, Weighill T, Scheffler K, Pond SLK. 2012. Detecting individual sites subject to episodic diversifying selection. PLoS Genet 8:e1002764. 10.1371/journal.pgen.1002764. 22807683 PMC3395634

[25] Murrell B, Moola S, Mabona A, Weighill T, Sheward D, Kosakovsky Pond SL, Scheffler K. 2013. FUBAR: a fast, unconstrained bayesian approximation for inferring selection. Mol Biol Evol 30:1196–1205. 10.1093/molbev/mst030. 23420840 PMC3670733

[26] Scheffler K, Martin DP, Seoighe C. 2006. Robust inference of positive selection from recombining coding sequences. Bioinformatics 22:2493–2499. 10.1093/bioinformatics/btl427. 16895925

[27] Kosakovsky Pond SL, Posada D, Gravenor MB, Woelk CH, Frost SDW. 2006. Automated phylogenetic detection of recombination using a genetic algorithm. Mol Biol Evol 23:1891–1901. 10.1093/molbev/msl051. 16818476

[28] Tsai Y, Holton T, Yeates TO. 2015. Diffusion accessibility as a method for visualizing macromolecular surface geometry. Protein Sci 24:1702–1705. 10.1002/pro.2752. 26189444 PMC4594669

