# Supplementary material for: HIV Coinfection Provides Insights for the Design of Vaccine Cocktails to Elicit Broadly Neutralizing Antibodies
Source: J Virol. 2022 Jun 27;96(14):e00324-22. doi: 10.1128/jvi.00324-22 (PMC9327685; doi:10.1128/jvi.00324-22)
Supplement: Supplemental file 1 — Fig. S1 to S4 and Table S1. Download jvi.00324-22-s0001.pdf, PDF file, 0.4 MB [file jvi.00324-22-s0001.pdf]

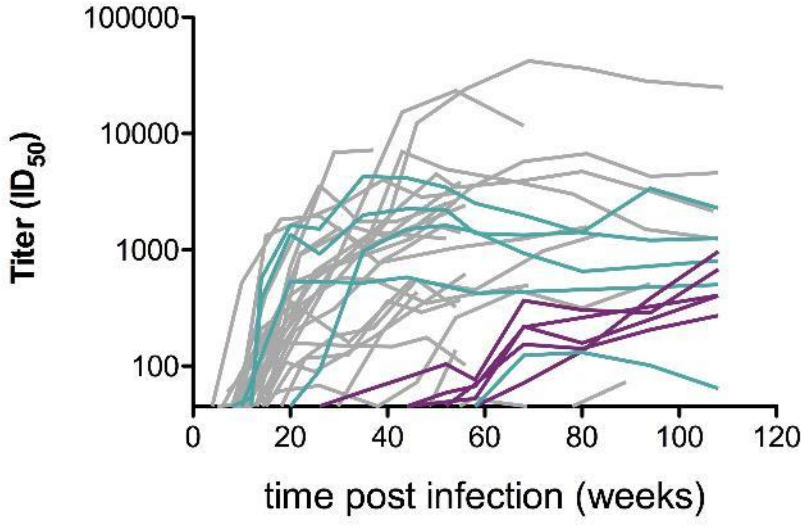

Supp Fig. 1 Weak autologous neutralizing antibody responses in CAP267. Autologous neutralizing antibody responses in CAP267 (teal and purple representing those to each variant respectively) are plotted alongside the neutralizing antibody responses to early/founder envelope clones in 23 other participants in the CAPRISA 002 cohort (grey). CAP267 neutralizing titers to several clones represent some of the lowest titer, and most delayed responses observed in the cohort, suggestive of immune interference in the antibody response.

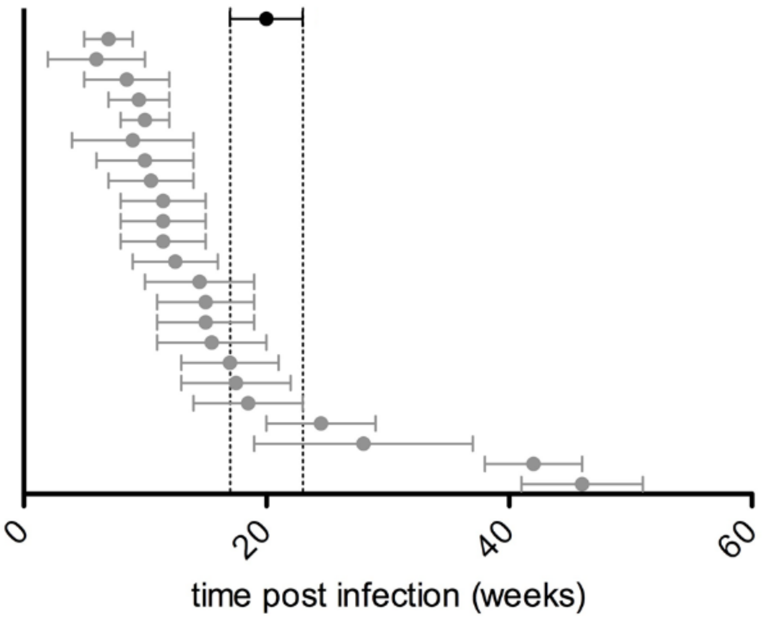

Supp Fig. 2 Neutralizing antibodies that arise to variant 2 in CAP137 are relatively delayed. Time from the inferred time of transmission to the detection of neutralizing antibody responses is shown for CAP137 variant 2 (black), compared to those against the early/founder variants in 23 other participants from the CAPRISA 002 acute infection cohort. Depicted is the midpoint and range between the last negative and first positive samples.

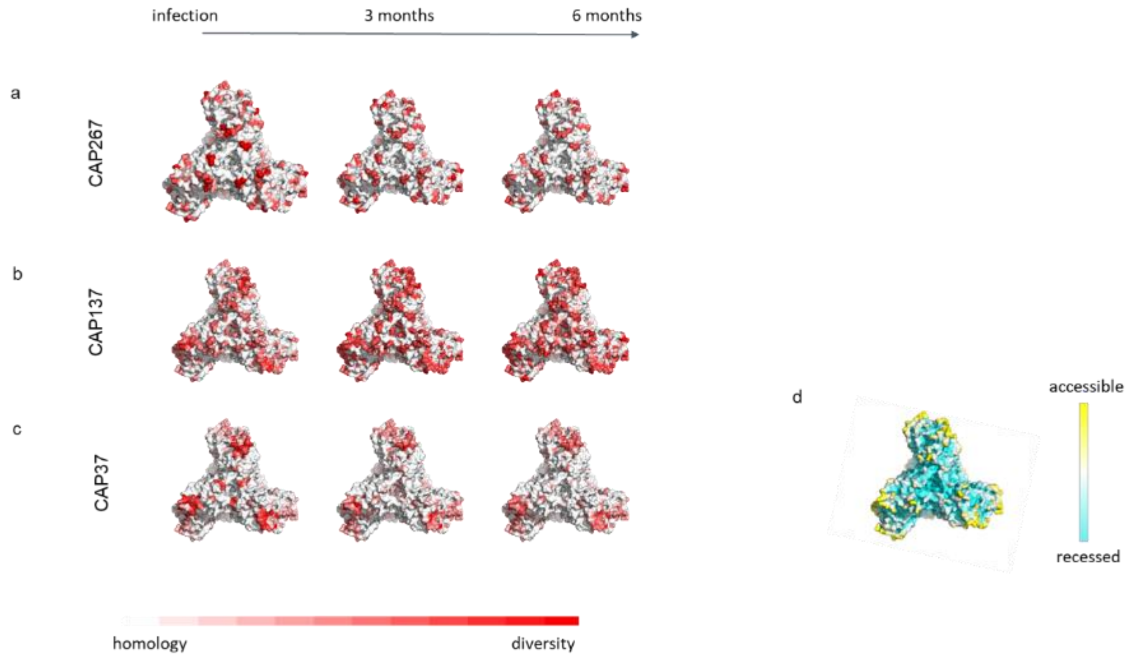

Supp. Fig 3. Summary of the Env diversity over time in early infection for the three individuals characterized here. a-c Depicted is the top-down view of the Env trimer (PDB:4ZMJ), coloured by entropy present in single genome sequences generated from each sample. **d** Accessibility, estimated by diffusion accessibility to the deglycosylated protein, of regions within the HIV Env trimer.

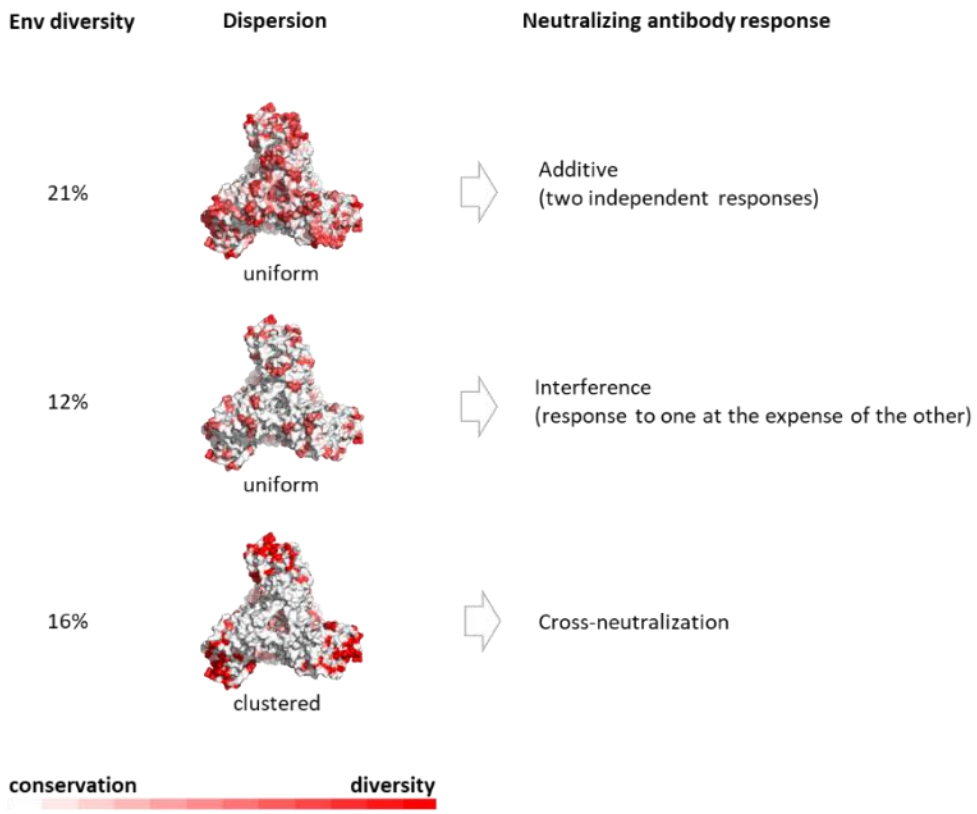

Supp Fig.4 **. Summary of the Env diversity and dispersion with associated outcomes for the three individuals characterized here**

		ID50									
		ConC			Du156			CAP45 G3			
		WT	N160A	K169E	WT	N160K	K169E	WT	N160A	K169E	MLV
CAP37	1.5 years	939	1089	1132	717	174	546	284	450	185	<40
	2 years	7991	1073	1018	745	2433	627	5619	1801	193	<40

Legend	
(dark red)	>2000
(orange)	200-2000
(yellow)	45-200
(white)	<40

		FOLD DIFFERENCE					
		Conc		Du156		CAP45 G3	
		N160A	K169E	N160K	K169E	N160A	K169E
CAP37	1.5 years	0.9	0.8	**4.1**	1.3	0.6	1.5
	2 years	**7.4**	**7.8**	0.3	1.2	**3.1**	**29.1**

Supp Table 1. Evidence of cross-reactive neutralizing antibodies targeting V2 in donor CAP37. Mutations in V2 significantly impact neutralization of heterologous viruses (ConC, Du156, CAP45.G3). ID_{50} titers are displayed in the top panel, and fold-change relative to wildtype viruses are shown in the bottom panel.
